# Assessing behavioural economic demand for morphine after an acute dose in individuals with- and without childhood trauma: a double-blind, randomised, controlled trial

**DOI:** 10.1007/s00213-025-06977-7

**Published:** 2025-12-04

**Authors:** Martin Trøstheim, Benjamin L. Berey, Elizabeth R. Aston, Matthew J. Gullo, Lee Hogarth, Graham Simpson, Rupert Broomby, Celia J. Morgan, Molly Carlyle

**Affiliations:** 1 Kongsberg Hospital, Vestre Viken Hospital Trust, Kongsberg, Norway; 2 Department of Psychology, University of Oslo, Forskningsveien 3A, Oslo, 0373 Oslo Norway; 3 Providence VA Medical Center, Rhode Island, USA; 4 Center for Alcohol and Addiction Studies, School of Public Health, Brown University, Providence, RI USA; 5 Department of Psychology, University of Queensland, Brisbane, Australia; 6 School of Applied Psychology, Griffith University, Brisbane, Australia; 7 Centre for Mental Health, Griffith University, Brisbane, Australia; 8 Department of Psychology, University of Exeter, Exeter, UK; 9 Anaesthesia & Pain Management, Royal Devon and Exeter NHS Foundation Trust, Exeter, UK; 10 Section for Trauma, Catastrophes and Forced Migration – Children and Youth, Norwegian Centre for Violence and Traumatic Stress Studies, Oslo, Norway; 11 Department of Psychiatryand Human Behavior, Brown University Alpert Medical School, Providence, RI USA

**Keywords:** Human, Stress, Trauma, Child maltreatment, Child neglect, Addiction, Substance abuse, Opioid, Motivation, Self-administration, Demand, Behavioural economics

## Abstract

**Background:**

Childhood trauma is associated with positive subjective responses to acute opioids in the laboratory. Yet, its impact on behavioural economic demand for opioids remains unclear. Because demand can predict future use and misuse, we investigated whether individuals with childhood trauma also display greater demand for opioids in the laboratory.

**Methods:**

This secondary analysis used data from a double-blind, randomised, controlled, counterbalanced trial. Across two sessions, individuals with (*n* = 26) and without (*n* = 21) childhood trauma received a high (0.15 mg/kg) and low (0.01 mg/kg) dose of intramuscular morphine 120 min before a hypothetical purchase task. Participants also regularly reported their subjective desire for the study drug. Area under the consumption and expenditure curves, and specific demand indices (*O*_*max*_, *P*_*max*_, *breakpoint*, *intensity*, *elasticity*), were analysed with non-parametric mixed models.

**Results:**

Consumption and expenditure for low dose morphine was lower in the childhood trauma group when compared to high dose morphine in this group, and low dose morphine in the control group (*p*s ≤ 0.03). Consistent results were obtained for specific demand indices *O*_*max*_, *P*_*max*_, and *breakpoint*, but not *intensity* or *elasticity*. Positive correlations between subjective desire and consumption and expenditure were only observed in the childhood trauma group (*Tau-c* = 0.24–0.34, *p*s ≤ 0.03).

**Conclusion:**

Under laboratory conditions, desires to use opioids among individuals with childhood traumas may not manifest in strong intentions to acquire these drugs. Diverging results from psychometric measures of subjective and economic opioid value highlight the need for future research to explore contextual and resilience factors to understand how problematic opioid use emerges after childhood traumas.

**Supplementary Information:**

The online version contains supplementary material available at https://doi.org/10.1007/s00213-025-06977-7.

## Introduction

Childhood traumas, such as abuse and neglect, are major life stressors that are associated with an increased risk of developing opioid use problems later in life [1–6], and are frequently reported by people with opioid use disorders (OUD) [7]. A heightened sensitivity to the rewarding subjective effects of opioids represents a candidate mechanism for the aetiological link between childhood trauma and the increased risk of OUD. This mechanism is supported by preclinical studies showing that rodents exposed to models of early life adversity such as maternal separation and limited resources) display greater opioid self-administration [8], conditioned place preference for opioid-paired locations [9], faster reinstatement of previously extinguished opioid-seeking behaviours, and higher demand (i.e., relative reinforcing value) for opioids [10]. However, few of these findings have been corroborated in human studies.

To our knowledge, only three studies have compared subjective responses to opioids in people with and without history of childhood trauma. One randomised controlled study examined subjective responses to acute intramuscular morphine administration in people who had or had not been exposed to severe childhood trauma [11]. This study found that when given a high dose of morphine (0.15 mg/kg), individuals with childhood traumas rated the drug effects as more subjectively likeable, felt more euphoric, reported greater desire to receive more of the drug, and experienced less adverse effects such as nausea and dizziness compared to individuals with no childhood traumas [11]. A subsequent observational study of patients who were administered remifentanil or oxycodone as part of routine care before surgery instead reported *lower* self-reported liking of the pre-surgical opioid in patients with greater childhood traumas [12]. The failure to conceptually replicate the original laboratory results could reflect state- or context-dependent effects arising from differences in the scenario in which the drug administration was offered in these studies (i.e., laboratory visit vs. impending surgery) [13], as well as the limited number of people with more severe adversities in the observational study. Lastly, a secondary analysis of data from a randomised placebo-controlled trial [14] yielded no conclusive evidence for a relationship between childhood trauma and subjective responses to buprenorphine in either direction [15]. A better understanding of the factors influencing an aetiological pathway between childhood trauma and susceptibility to opioid misuse is needed to improve clarity on why some people with history of childhood trauma develop persistent opioid use.

People with existing opioid use problems are more likely to retrospectively report positive initial experiences with opioid drugs [16], and subjective ratings of rewarding drug effects have been considered the gold standard for determining the potential for misuse [17, 18]. Yet, there is little research on whether these initial positive experiences are associated with an increased decision to seek out, obtain, and consume opioids in the early stages of drug use. Such decision making has been measured frequently in behavioural economic research, where drug use decisions in the context of economic costs (i.e., demand) are assessed by hypothetical purchase tasks [19]. In these tasks, respondents indicate the number of units of the commodity they would purchase at increasing hypothetical unit prices. Results from prior systematic reviews and meta-analyses provide evidence for reliable, moderate-sized correlations between behavioural economic demand for various substances (e.g., alcohol, cannabis, cocaine) and measures of drug use frequency, quantity, and severity [20, 21].

Although frequently employed in studies of demand for licit drugs with known market prices such as alcohol and tobacco, the hypothetical purchase task has also been used to study demand for restricted drugs, including opioids, that are being sold on the illicit market at varying street prices [20]. Studies of opioid demand have however been restricted to people with opioid use disorder who are familiar with both the subjective effects and the street prices of the drugs [22–27]. It is therefore unclear if initial subjective responses to opioids translate into behavioural economic demand for these drugs in people who experience the positive drug effects, yet do not have a history of drug use problems. Moreover, no study to date has evaluated opioid demand in people with history of childhood trauma—a population known to be at risk of developing problematic opioid use.

Here, we analysed hypothetical purchase task data collected as part of a previously reported laboratory study to determine whether behavioural economic demand for the opioid morphine differs between people with and without history of childhood trauma [11]. Given this previous finding of increased self-reported liking, euphoria, and desire for morphine in individuals with childhood traumas in a laboratory setting, and the well-established link between such responses and risk of opioid misuse [17, 18], we expected the childhood trauma-exposed group to also display increased behavioural economic demand for morphine in the same setting. We also explored the correlation between self-reported desires and behavioural economic demand for morphine to evaluate whether these desires and spending intentions converge. By combining methods from the fields of experimental psychopharmacology and behavioural economics, this study may reveal which psychometric measures are superior at detecting a difference in opioid value between individuals with and without childhood traumas. It may also provide preliminary evidence for a risk pathway from initial opioid exposure to future problematic opioid use.

## Methods

### Participants

The analyses presented here used data from secondary outcome measures collected during the previously reported PROACT study (IRAS ID 240232) [11]. This study took place at the NIHR Exeter Clinical Research Facility at the Royal Devon and Exeter Hospital and recruited 52 mentally and physically healthy volunteers (35 female, mean age ± SD = 30.90 ± 14.90 years). Following a rigorous screening procedure [11], volunteers were included if they reported either no history of childhood trauma (*n* = 25), or history of severe childhood trauma (*n* = 27) on at least one of the subscales (i.e., emotional or physical abuse or neglect, or sexual abuse before the age of 18) from the 25-item Childhood Trauma Questionnaire (CTQ) [28]. Volunteers with contraindications for morphine, history of mental disorders, or history of alcohol or substance use disorders were excluded (described in greater detail in the main study publication [11]. The study was reviewed by the NHS Research Ethics Committee (Wales REC 4 reference 18/WA/0098) and conducted in accordance with the Declaration of Helsinki. All participants gave written, witnessed, informed consent and were compensated with £50 for participating.

### Design

This was a double-blind, randomised, controlled, counterbalanced crossover study in which participants completed two sessions that lasted 3.5–4.5 h each and were held 7 ± 1 days apart. During each session, participants received either a high dose of morphine (0.15 mg/kg, maximum 10 mg) or a very low dose control (0.01 mg/kg, i.e., a miniscule dose with no subjective effects) intramuscularly. A low-dose control containing negligible amounts of morphine was preferred over a pure placebo to better conceal the randomisation, as participants were told they would receive two different doses of morphine. Drug order was counterbalanced and randomised on a computer. The study drugs were prepared with saline by an anaesthetist using identical, unlabelled 2 ml syringes and injected into the anterolateral thigh muscle. The participants, experimenters and nurses, but not the anaesthetists who administered the drugs, were blind to the contents of the syringes.

### Measures

#### Demographics and clinical characteristics

We collected demographic and clinical information about the participants, including sex, age, BMI, and prior opioid experience. In addition to the CTQ [28], participants completed the 21-item Depression Anxiety Stress Scales (DASS-21) [29], the 12-item Multidimensional Scale of Perceived Social Support (MSPSS) [30], and the 13-item Pain Catastrophizing Scale (PCS) [31].

#### Self-reported drug wanting

**Fig. 1:**
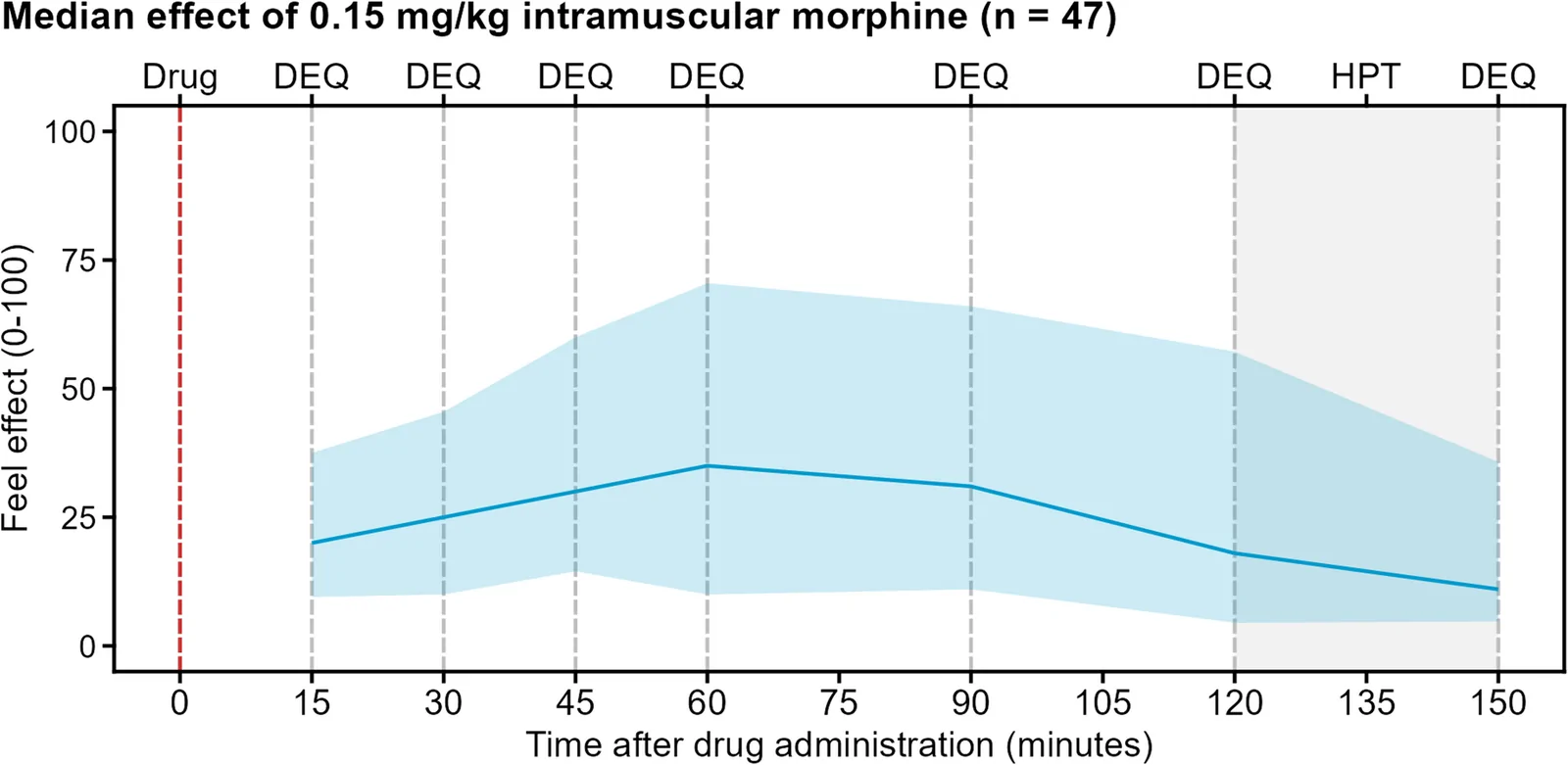
Session timeline showing the time points for outcome assessments (dashed vertical lines) in relation to the median subjective effect (i.e. *Feel effect*; blue solid line) for the high dose (0.15 mg/kg) of intramuscular morphine across all participants included in the analyses (*n* = 47). The grey shaded area indicates the time frame during which participants completed the HPT. The blue ribbon indicates the interquartile range. See Fig. 1 in Carlyle et al. [11] for the complete study timeline

We used the Drug Effects Questionnaire (DEQ) [32] to measure participants’ self-reported wanting for the morphine dose (“Would you like *MORE* of the drug you took, right now?”) on a 0–100 visual analogue scale (VAS; 0 = “not at all”, 100 = “extremely”) 15, 30, 45, 60, 90, 120 and 150 min after drug administration (Fig. 1). Responses were collected using a touch-screen iPad. Results for this outcome measure have been reported previously [11]. To summarise participants’ self-reported drug wanting across assessment time points leading up to the hypothetical purchase task, we computed the percentage of the total possible area under the curve for the VAS ratings (*Want more*) made from 15 to 120 min after drug administration.

#### Hypothetical morphine purchase task (HPT)

Between 120 and 150 min after drug administration (Fig. 1), participants completed a hypothetical purchase task (**Supplemental material [SM] 1**). This task was adapted from a cigarette purchase task [33]. The HPT instructed participants to imagine that they could purchase the dose of morphine they received earlier in the session across 41 escalating prices ranging from £0–5 in non-uniform increments (£0.02 increments from £0–0.6.6, £0.1 increments from £0.6–1.6, £0.5 increments from £1–3, and £1 increments from £3–5). Prices were not changed from the original questionnaire because the willingness to purchase morphine in the absence of medical need in the current study population was unknown, but expected to be lower than in populations with opioid use problems. Participants were not told the cost of the dose they had received previously in the session, and were not provided with the UK street price of morphine (~£4.20 per 10 mg [34]). The HPT also asked participants to imagine that (1) their income remained unchanged, and (2) they did not have access to any other pain medications. There was no upper limit to the number of doses they could purchase. No timeframe was provided for when they had to consume the doses. Participants completed the HPT on pen and paper.

Several outcome measures were derived from the responses to the HPT. As general measures of consumption (i.e., additional doses) and expenditure (i.e., consumption multiplied by price) across prices, we calculated the areas under the consumption curve (*Consumption*_*AUC*_) and expenditure curve (*Expenditure*_*AUC*_) using the trapezoidal rule [35]. To further probe participants’ economic demand behaviour, we computed the specific demand indices *Intensity* (i.e., the consumption when additional doses are free), *O*_*max*_ (i.e., the maximum expenditure on additional doses), *P*_*max*_ (i.e., the price that maximises expenditure), *Breakpoint* (i.e., the lowest price that suppresses consumption of additional doses), and *Elasticity* (i.e., the rate of change in consumption of additional doses as price increases; **SM2**) [36].

### Statistical analyses

All data processing and analysis was performed in R [37] (**SM4**). HPT data quality was evaluated by checking for increasing trends (i.e., an overall increase in consumption across the increasing price range), bounces (i.e., an increase in existing consumption with a single increase in price), and reversals from zero (i.e., an increase from zero to non-zero consumption with a single increase in price) [38]. Due to the small sample size, skewed distributions, and considerable number of extreme outliers (reported in **SM3**), parametric models would likely overestimate the typical self-reported drug wanting, consumption, expenditure, and demand indices in this sample (**SM3**). We therefore used non-parametric, rank-based approaches for all analyses [39]. These analyses are minimally impacted by outliers, as extreme values receive a rank that is only one point higher than the closest ranking value. Outliers were therefore kept in the rank-based non-parametric analyses and remained unadjusted to ensure that participants would retain their original ranks on outcome measures. Rank ties were accounted for in all of the non-parametric tests. An overview of how rank scores and original values correspond for each outcome measure is available in **SM5**.

Group differences in sample characteristics were assessed with Kruskal-Wallis *H*-tests [40] and Fisher’s exact tests [41, 42].

To test the effects of childhood trauma and morphine dose on the overall measures of consumption and expenditure (primary analyses), and the specific demand indices (secondary analyses), we fitted rank-based non-parametric mixed models and computed ANOVA-type statistics (*ATS*) [43, 44]. These models included *Group* (childhood trauma vs. control), *Dose* (high dose morphine vs. low dose morphine), and the *Group*×*Dose* interaction as predictors and were run separately for the outcomes *Consumption*_*AUC*_ and *Expenditure*_*AUC*_ (primary analyses), *Intensity*, *O*_*max*_, *P*_*max*_, *Breakpoint*, and *Elasticity* (secondary analyses). To account for multiple testing, we adjusted *p*-values with the Bonferroni method based on the number of outcomes in each analysis (primary analyses: *n* = 2; secondary analyses: *n* = 5 outcomes). Similar non-parametric models were used to verify that the previously observed elevated desires (*Want more*) for high dose morphine [11] replicated in the subsample with valid HPT data (reported in **SM9**).

Relative treatment effects (RTE) were calculated for each *Group*×*Dose* combination and indicated the probability that a randomly drawn observation from the entire sample ranked lower on the outcome than a randomly drawn observation from the *Group×Dose* combination for which the RTE was estimated [44].

Post-hoc rank-based pairwise comparisons with Bonferroni correction (*n* = 4 comparisons) were conducted with non-parametric repeated measures models for comparisons between doses within groups [43, 44], and with Kruskal-Wallis *H*-tests for comparisons between groups within doses [40].

Sensitivity analyses used rank-based non-parametric mixed models [43, 44] to separately test the effects of dose order and prior opioid experience on self-reported drug wanting, consumption, expenditure, and demand indices.

To explore the correspondence between self-reported drug wanting (*Want more*) and consumption (*Consumption*_*AUC*_), expenditure (*Expenditure*_*AUC*_), and demand indices (*Intensity*, *O*_*max*_, *P*_*max*_, *Breakpoint*, and *Elasticity*), we computed Stuart-Kendall *Tau-c* rank-based correlations as this statistic accounts for differences in scale resolution between outcomes [45].

## Results

### Sample characteristics and exclusions

Fifty-two people participated in the study and completed both dose sessions. Four controls were excluded from the analyses due to missing responses to the HPT in one of the sessions, which prevented calculation of demand indices. One participant with childhood trauma was excluded due to high inconsistency in responses to the HPT (i.e., 8 bounces) in one session. The final analytic sample consisted of 47 participants: 26 with childhood trauma and 21 controls. Sample characteristics are available in Table 1.

**Table 1:** Descriptive statistics for sample characteristics.

	Control (*n* = 21)	Childhood trauma (*n* = 26)	
Sex			*p* = 0.77
Women	14 (67%)	16 (62%)	
Men	7 (33%)	10 (38%)	
Age (years)			*p* = 0.26
Mean (SD)	33.90 (16.55)	29.12 (13.61)	
Median (IQR)	26.00 (22.00–40.00)	24.50 (20.00–29.75)	
Range	19.00–65.00	18.00–65.00	
BMI (kg/m^2^)			*p* = 0.40
Mean (SD)	27.99 (24.56)	24.78 (4.63)	
Median (IQR)	22.60 (20.78–24.63)	23.56 (21.12–28.04)	
Range	19.52–134.74	19.16–34.76	
MSPSS (1–7)			*p* < 0.0001
Mean (SD)	6.25 (0.70)	4.74 (1.23)	
Median (IQR)	6.42 (6.00–6.75)	4.62 (3.88–5.65)	
Range	4.33–7.33	2.50–7.50	
DASS Depression (0–21)			*p* < 0.01
Mean (SD)	0.90 (1.14)	4.58 (5.10)	
Median (IQR)	0.00 (0.00–2.00)	2.50 (0.25–8.25)	
Range	0–4	0–17	
DASS Anxiety (0–21)			*p* = 0.02
Mean (SD)	0.95 (1.53)	3.08 (3.63)	
Median (IQR)	0.00 (0.00–1.00)	1.5 (0.25–4.75)	
Range	0–6	0–11	
PCS (0–52)			*p* < 0.001
Mean (SD)	10.14 (7.22)	18.81 (8.53)	
Median (IQR)	11.00 (5.00–13.00)	19.00 (12.25–25.75)	
Range	0–24	4–39	
CTQ (25–125)			*p* < 0.0001
Mean (SD)	27.52 (2.14)	63.96 (13.68)	
Median (IQR)	27.00 (26.00–29.00)	60.00 (54.00–70.50)	
Range	25.00–32.00	00–100.00	
Prior opioid experience			*p* = 0.24
Yes	7 (33%)	14 (54%)	
No	14 (67%)	12 (46%)	
Dose order			*p* = 1
Low dose-High dose	12 (57%)	14 (54%)	
High dose-Low dose	9 (43%)	12 (46%)	

*BMI* Body mass index, *MSPSS* Multidimensional Scale of Perceived Social Support, *DASS* Depression Anxiety Stress Scales, *PCS* Pain Catastrophizing Scale, *CTQ* Childhood Trauma Questionnaire, *SD* standard deviation, *IQR* interquartile range

Prior opioid experience was measured by asking participants whether they had ever been medically administered morphine or other opioids in the past, or whether they taken non-medical opioids recreationally in the past (all required a yes/no response; **SM6**). Differences between groups in sex, prior opioid experience, and dose order were assessed with Fisher’s exact tests, while differences between groups in age, BMI, MSPSS, DASS Depression, DASS Anxiety, PCS, and CTQ were assessed with Kruskal-Wallis H-tests

### Primary analyses: overall consumption and expenditure

**Fig. 2:**
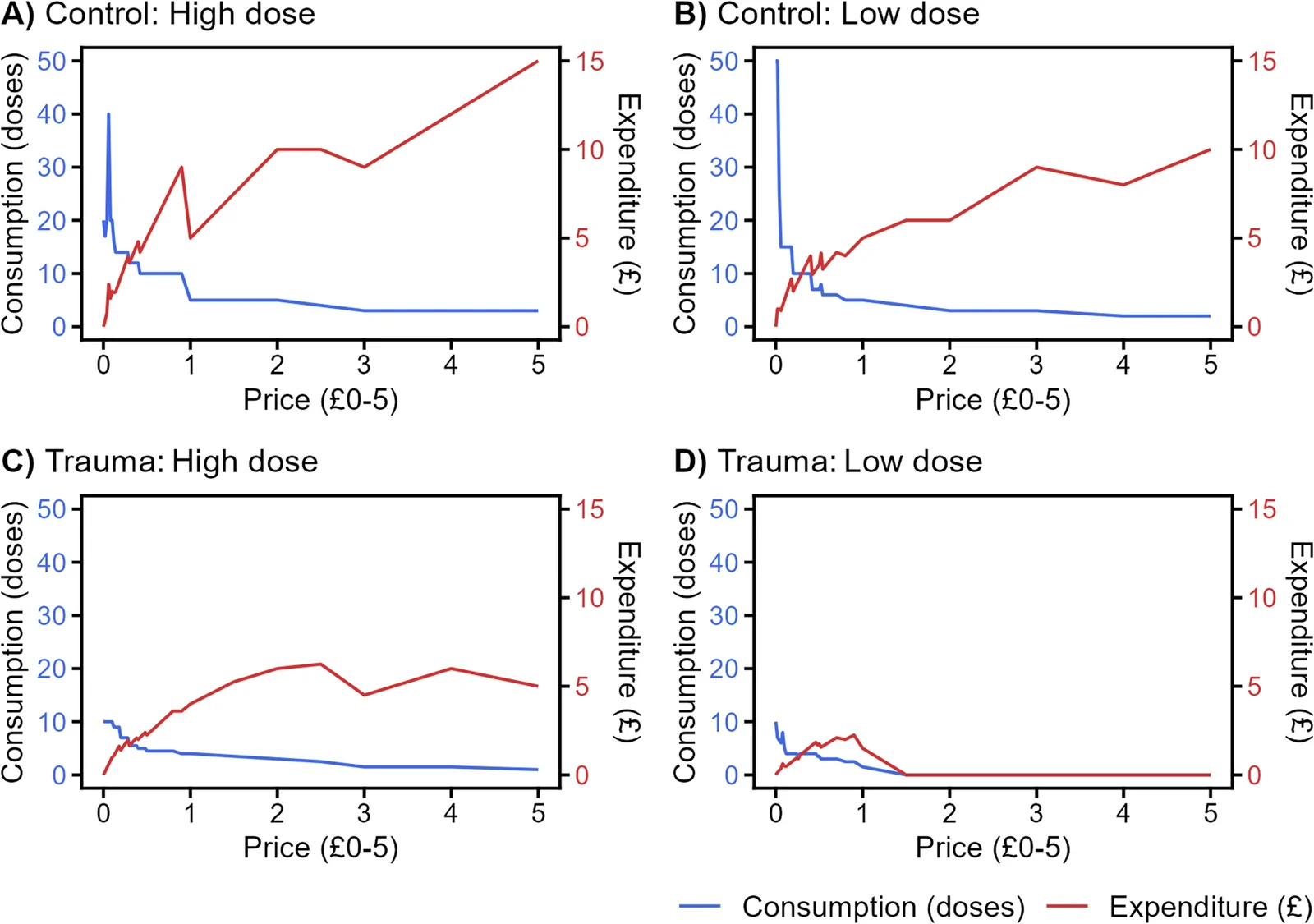
Untransformed empirical consumption and expenditure curves. Lines indicate the median consumption in number of additional doses (blue) and expenditure in £ (red) at each price point in the HPT

Hypothetical consumption of and expenditure on additional doses of high dose and low dose morphine across price points in participants with and without childhood trauma are presented in Fig. 2. There were few inconsistencies (i.e., occurrences of increasing consumption with increasing price) in participants’ responses to the HPT (**SM8**). Descriptive statistics for *Consumption*_*AUC*_ and *Expenditure*_*AUC*_ are reported in Table 2.

**Table 2:** Descriptive statistics for drug dose, drug consumption and expenditure, drug demand indices, and self-reported drug wanting leading up to the HPT

	Control (*n* = 21)		Childhood trauma (*n* = 26)	
	High dose	Low dose	High dose	Low dose
Morphine dose (mg)				
Mean (SD)	9.11 (0.94)	0.65 (0.13)	9.44 (0.75)	0.72 (0.15)
Median (IQR)	9.43 (8.40–10.00)	0.63 (0.56–0.71)	9.97 (8.83–10.00.83.00)	0.69 (0.59–0.84)
Range	7.29–10.00.29.00	0.49–0.99	7.89–10.00.89.00	0.53–0.99
Want more (0–100)				
Mean (SD)	7 (12)	7 (13)	40 (31)	19 (22)
Median (IQR)	0 (0–7)	0 (0–7)	48 (4–62)	9 (0–38)
Range	0–44	0–50	0–91	0–66
Consumption_AUC_				
Mean (SD)	141 (207)	255 (479)	44 (73)	20 (33)
Median (IQR)	25 (10–160)	16 (10–301)	17 (7–42)	7 (0–20)
Range	0–660	0–1648	0–336	0–132
Expenditure_AUC_				
Mean (SD)	192.06 (289.73)	229.65 (385.60)	51.09 (66.30)	24.20 (42.90)
Median (IQR)	40.27 (4.50–221.05)	32.95 (6.47–192.40)	24.98 (6.88–61.46)	2.68 (0.00–21.06)
Range	0–930.75	0–1356.09	0–263.89	0–141.61
Intensity (doses)				
Mean (SD)	2530 (10886)	5137 (21757)	75 (199)	43 (104)
Median (IQR)	20 (5–100)	50 (6–100)	10 (4–82)	10 (0–30)
Range	0–50000	0–100000	0–1000	0–500
O_max_ (£)				
Mean (SD)	56.63 (85.20)	99.05 (177.34)	31.78 (62.60)	8.90 (13.45)
Median (IQR)	15.00 (7.50–56.00)	10.00 (3.20–108.00)	10.00 (5.00–25.00)	3.37 (0.00–9.88)
Range	0.00–325.00.00.00	0.00–700.00	0.00–282.80	0.00–50.00
P_max_ (£)				
Mean (SD)	2.73 (1.90)	2.09 (2.02)	2.34 (2.12)	1.23 (1.72)
Median (IQR)	3.00 (0.90–5.00.90.00)	1.50 (0.24–4.00)	2.00 (0.30–5.00)	0.31 (0.00–1.88)
Range	0.00–5.00	0.00–5.00	0.00–5.00	0.00–5.00
Breakpoint (£)				
N	6 (29%)	7 (33%)	9 (35%)	19 (73%)
Mean (SD)	0.69 (0.96)	1.36 (1.64)	0.80 (1.10)	1.02 (1.35)
Median (IQR)	0.33 (0.08–0.84)	0.28 (0.12–2.50)	0.30 (0.00–1.50)	0.18 (0.00–1.75)
Range	0.00–2.50	0.00–4.00	0.00–3.00	0.00–4.00
Elasticity				
Mean (SD)	0.69 (1.00)	0.87 (0.87)	0.61 (0.57)	0.66 (0.80)
Median (IQR)	0.38 (0.14–0.82)	0.69 (0.36–0.99)	0.54 (0.15–0.91)	0.36 (0.00–1.11)
Range	0–4.29	0–3.24	0–1.94	0–3.27

*SD* standard deviation, *IQR* interquartile range

Self-reported drug wanting (*Want more*) was rated on a 0-100 VAS at 15, 30, 45, 60, 90, and 120 minutes after drug administration and is expressed as the percentage of the total possible AUC (10500). For other self-reported drug effects, see **SM7**.

#### Consumption

**Fig. 3:**
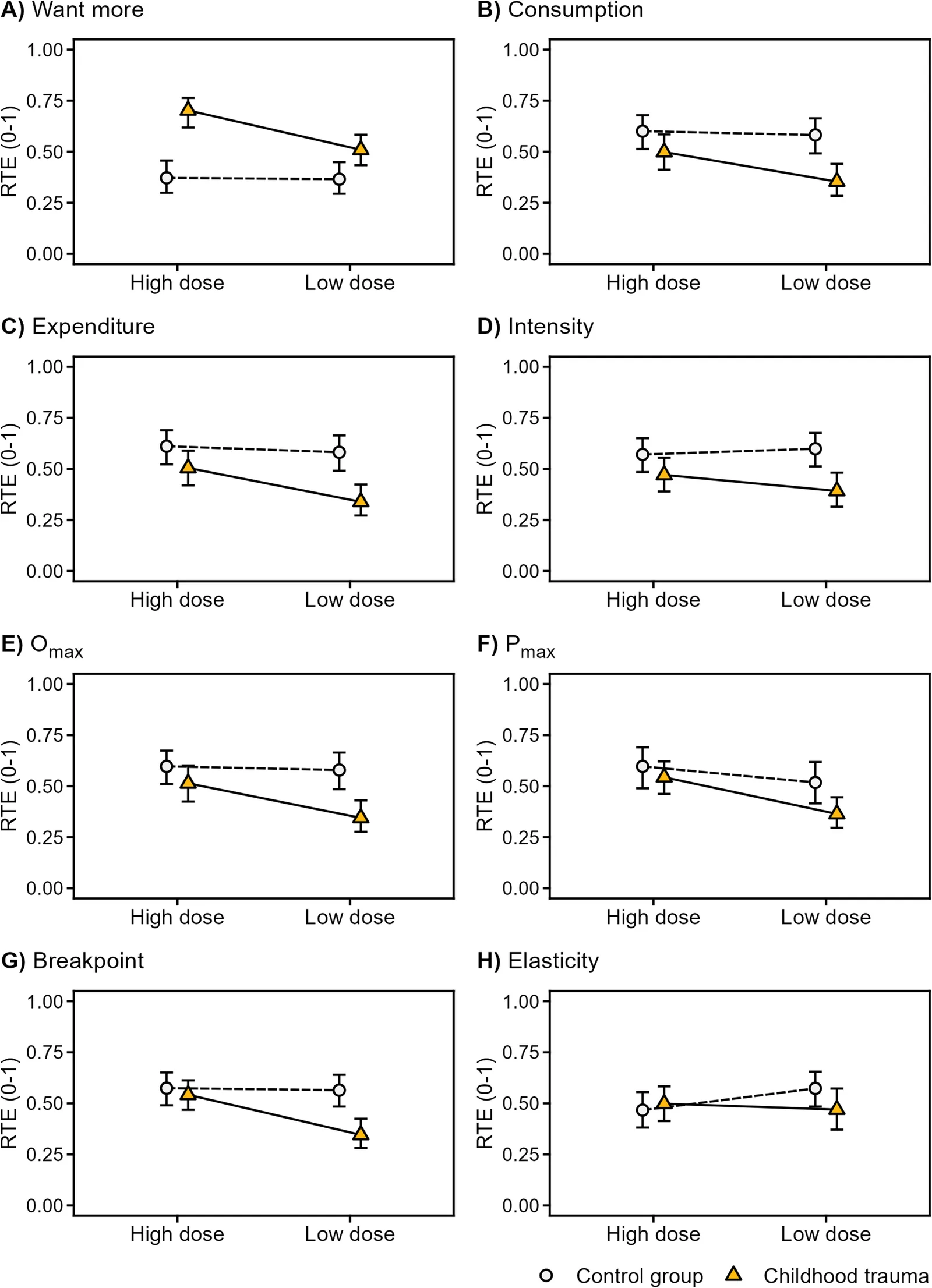
Relative treatment effects (RTE) for *Want more*, *Consumption*_*AUC*_, *Expenditure*_*AUC*_, *Intensity*, *O*_*max*_, *P*_*max*_, *Breakpoint*, and *Elasticity*. The RTE indicates the probability that a randomly drawn observation from the entire sample has lower *Want more*, *Consumption*_*AUC*_, *Expenditure*_*AUC*_, *Intensity*, *O*_*max*_, *P*_*max*_, *Breakpoint*, or *Elasticity* than a randomly drawn observation from the particular *Group*×*Dose* combination for which the RTE was estimated. Error bars indicate 95% confidence intervals. See **SM10** for exact RTE values

There was a significant main effect of *Dose* (*ATS*_1_ = 7.12, *p*_adjusted_ = 0.02), but no significant main effect of *Group* (*ATS*_1_ = 4.69, *p*_adjusted_ = 0.06) or *Group*×*Dose* interaction effect (*ATS*_1_ = 4.23, *p*_adjusted_ = 0.08) on *Consumption*_*AUC*_. As indicated by post-hoc pairwise comparisons, there was less *Consumption*_*AUC*_ for low dose morphine (Kruskal-Wallis *H*_1_ = 6.98, *p*_adjusted_ = 0.03), but not high dose morphine (Kruskal-Wallis *H*_1_ = 1.91, *p*_adjusted_ = 0.67), in the childhood trauma group than in the control group (Fig. 3A). Likewise, there was less *Consumption*_*AUC*_ for low dose morphine than high dose morphine in the childhood trauma group (*ATS*_1_ = 7.00, *p*_adjusted_ = 0.03), but not in the control group (*ATS*_1_ = 0.46, *p*_adjusted_ = 1; Fig. 3A). Sensitivity analyses indicated no significant *Dose order* main effect or *Dose order*×*Dose* interaction effect on *Consumption*_*AUC*_, and no significant *Prior opioid experience* main effect or *Prior opioid experience*×*Dose* interaction effect on *Consumption*_*AUC*_ (all *p*s > 0.05; **SM11-12**).

#### Expenditure

For *Expenditure*_*AUC*_, there were significant main effects of *Group* (*ATS*_1_ = 5.33, *p*_adjusted_ = 0.04) and *Dose* (*ATS*_1_ = 10.50, *p*_adjusted_ < 0.01), and a significant the *Group*×*Dose* interaction effect (*ATS*_1_ = 5.09, *p*_adjusted_ < 0.05). Post-hoc pairwise comparisons indicated less *Expenditure*_*AUC*_ on low dose morphine (Kruskal-Wallis *H*_1_ = 8.12, *p*_adjusted_ = 0.02), but not high dose morphine (Kruskal-Wallis *H*_1_ = 2.03, *p*_adjusted_ = 0.62), in the childhood trauma group than in the control group (Fig. 3B). *Expenditure*_*AUC*_ on low dose morphine was less than *Expenditure*_*AUC*_ on high dose morphine in the childhood trauma group (*ATS*_1_ = 10.48, *p*_adjusted_ < 0.01), but not in the control group (*ATS*_1_ = 0.87, *p*_adjusted_ = 1; Fig. 3B). There was no significant *Dose order* main effect, *Dose order*×*Dose* interaction effect, *Prior opioid experience* main effect, or *Prior opioid experience*×*Dose* interaction effect on *Expenditure*_*AUC*_ in the sensitivity analyses (all *p*s > 0.05; **SM11-12**).

### Secondary analyses: demand indices

Descriptive statistics for demand indices are reported in Table 2. The pattern of results for individual demand indices tended to correspond to those obtained for overall consumption and expenditure (Fig. 3). For *Intensity* (i.e., consumption at £0), there were no significant main effect of *Group* (*ATS*_1_ = 3.94, *p*_adjusted_ = 0.24) or *Dose* (*ATS*_1_ = 0.79, *p*_adjusted_ = 1) or *Group*×*Dose* interaction (*ATS*_1_ = 3.48, *p*_adjusted_ = 0.31). For *O*_*max*_ (i.e., maximum expenditure), the *Dose* main effect (*ATS*_1_ = 7.65, *p*_adjusted_ = 0.03), but not the *Group* main effect (*ATS*_1_ = 4.55, *p*_adjusted_ = 0.16) or *Group*×*Dose* interaction effect (*ATS*_1_ = 5.02, *p*_adjusted_ = 0.13), was significant. Likewise, there was a significant *Dose* main effect (*ATS*_1_ = 8.43, *p*_adjusted_ = 0.02), but no significant *Group* main effect (*ATS*_1_ = 2.35, *p*_adjusted_ = 0.63) or *Group*×*Dose* interaction effect (*ATS*_1_ = 1.29, *p*_adjusted_ = 1) on *P*_*max*_ (i.e., price associated with maximum expenditure).

There was no significant main effect of G*roup* (*ATS*_1_ = 3.67, *p*_adjusted_ = 0.28) on *Breakpoint* (i.e., price that suppresses consumption to zero), but instead significant main effects of *Dose* (*ATS*_1_ = 11.63, *p*_adjusted_ < 0.01) and the G*roup*×*Dose* interaction effect (*ATS*_1_ = 9.56, *p*_adjusted_ < 0.01). Finally, neither the *Group* main effect (*ATS*_1_ = 0.23, *p*_adjusted_ = 1), *Dose* main effect (*ATS*_1_ = 1.10, *p*_adjusted_ = 1), nor the G*roup*×*Dose* interaction effect (*ATS*_1_ = 3.33, *p*_adjusted_ = 0.34) on *elasticity* (i.e., rate of change in consumption) was significant.

Post-hoc pairwise comparisons indicated lower *O*_*max*_, *P*_*max*_, and *Breakpoint*, but not *Intensity* or *Elasticity*, for low dose morphine in the childhood trauma group when compared to high dose morphine in the same group and/or low dose morphine in the control group (**SM10**). Sensitivity analyses indicated no significant *Dose order* main effect, *Dose order*×*Dose* interaction effect, *Prior opioid experience* main effect, or *Prior opioid experience*×*Dose* interaction effect on any of the demand indices (all *p*s ≥ 0.05; **SM11-12**).

### Exploratory analyses: correlations between self-reported drug wanting and consumption, expenditure, and demand indices

The correspondence between self-reported drug wanting (*Want more*) leading up to the HPT and drug consumption (*Consumption*_*AUC*_), expenditure (*Expenditure*_*AUC*_), and demand indices (*Intensity*, *O*_*max*_, *P*_*max*_, *Breakpoint*, and *Elasticity*) was assessed using rank-based correlations. Significant positive correlations were observed in the trauma group only (Table 3).

**Table 3:** Rank-based correlations (Stuart-Kendall Tau-c) between want more leading up to the HPT and drug consumption, expenditure, and demand indices

	Control (*n* = 21)		Childhood trauma (*n* = 26)	
	High dose	Low dose	High dose	Low dose
Consumption_AUC_	−0.03 (*p* = 0.85)	0.08 (*p* = 0.62)	0.34 (*p* < 0.01)	0.25 (*p* = 0.04)
Expenditure_AUC_	0.01 (*p* = 0.95)	0.10 (*p* = 0.50)	0.32 (*p* < 0.01)	0.24 (*p* = 0.04)
Intensity	0.02 (*p* = 0.92)	0.17 (*p* = 0.30)	0.38 (*p* < 0.001)	0.11 (*p* = 0.38)
O_max_	0.02 (*p* = 0.90)	0.16 (*p* = 0.33)	0.32 (*p* < 0.01)	0.26 (*p* = 0.03)
P_max_	0.11 (*p* = 0.37)	−0.09 (*p* = 0.53)	0.10 (*p* = 0.44)	0.36 (*p* < 0.01)
Breakpoint	0.02 (*p* = 0.86)	0.06 (*p* = 0.59)	0.18 (*p* = 0.13)	0.33 (*p* < 0.01)
Elasticity	0.06 (*p* = 0.68)	0.11 (*p* = 0.39)	0.08 (*p* = 0.51)	0.03 (*p* = 0.84)

All *p*-values are unadjusted

## Discussion

People with history of childhood traumas are at increased risk of developing opioid use disorder [4]. Key indicators of opioid misuse potential include positive subjective responses to acute opioid administration [17], such as self-reported desire to use opioids again. We have previously shown that people with childhood traumas report increased wanting for a high dose of morphine following acute administration of this opioid in a laboratory setting [11]. The current analyses used hypothetical purchase task data collected in the same double-blind, randomised, low dose-controlled counterbalanced crossover study to investigate whether these individuals also displayed greater behavioural economic demand for additional high dose morphine.

Contrary to our expectations, we observed no differences in hypothetical consumption of and expenditure on high dose morphine (0.15 mg/kg) between people with and without childhood traumas. Greater subjective desire for high dose morphine in people with childhood traumas did therefore not translate to greater intentions to purchase and consume this drug again. For low dose morphine (0.01 mg/kg), we reported decreased consumption and expenditure in participants with childhood traumas. When inspecting the specific demand indices, we found that people with childhood traumas reported lower demand for low vs. high dose morphine on three key indices: *O*_*max*_—the maximum expenditure on additional doses; *P*_*max*_—the price that maximises expenditure; and *Breakpoint*—the lowest price that suppresses demand. However, *Intensity* (consumption when additional doses are free) and *elasticity* (the rate of change in consumption as price increases) remained unaffected. While consumption and expenditure declined at comparable rates as price increased for both morphine doses in individuals with childhood trauma, demand for the lower dose was suppressed at lower prices. This may indicate that participants with childhood traumas found the low dose less reinforcing, and subsequently reported lower demand for this commodity.

In the exploratory analyses we observed positive correlations between self-reported wanting and behavioural economic demand for either dose of morphine in the childhood trauma group, suggesting that these individuals’ intentions to purchase morphine were informed by self-reported desires to receive more of this opioid throughout the sessions. We did not, however, find conclusive support for this association in individuals without childhood trauma, whom displayed comparable demand across low and high dose morphine irrespective of whether it produced desirable subjective effects. A tentative connection between desires and intentions among participants with, but not without, childhood traumas could help explain why the former group reported reduced demand for low dose morphine (i.e., because it produced minimal subjective effects [11] and is therefore deemed as less desirable). Another explanation could be that prior experience with opioids helped identification of and/or altered expectations about the drug effects, as prior opioid experience is associated with increased likelihood of positive subjective responses in the future [46]. Just under half (45%) of the participants in the sample had reported prior (minimal) opioid experience. However, the frequency of prior opioid experience did not significantly differ between the childhood trauma and non-trauma groups (**SM6**). We did not assess the quality of these past experiences, nor did we assess participants’ expectations about opioid effects. Participants with childhood trauma—who also reported greater depression, anxiety, and pain catastrophising symptoms—may have had more positive prior experiences of expectations of the analgesic, sedative, anxiolytic and antidepressive effects of opioids [47]. Nonetheless, that morphine was delivered in an undisclosed dose (either low or high) prior to the hypothetical purchasing task (HPT), and that participants were instructed to indicate their demand based on their experience within the session, likely minimised the influence of prior experiences and expectations on the results. Future studies should address the quality of prior opioid experiences and how these influence expectations about future drug effects, as well as how acute opioid administration affects mood, stress and pain, to better understand the motivations for acquiring opioids in people with history of childhood trauma.

Despite the difference in depression and anxiety symptoms, both participants with and without childhood traumas reported symptom severity within the normal-to-mild range [48]. Moreover, none of the participants in the childhood trauma group presented with major psychiatric disorders or problematic opioid use, despite reporting high severity on one or more subscales for childhood abuse or neglect, and therefore being at an increased risk of developing mental health problems [4, 49]. This resilience may extend to reduce opioid demand and, in turn, help participants with childhood trauma in this study resist the desire to acquire additional high doses of morphine which had previously elicited pleasurable effects [11]. Future studies should investigate how resilience factors such as optimism, cognitive flexibility, active coping, access to and enjoyment from alternative substance-free reinforcers (e.g., social support), prior psychological intervention/support, and physical activity influence responses to and demand for opioid drugs in people with childhood traumas to help understand why some individuals do and do not develop problematic use [50].

Exploratory analyses suggested a small yet significant association where individuals with higher anxiety and pain-catastrophising (both of which were higher for people in the childhood trauma group) reported a higher breakpoint for the high dose of morphine (**SM13**). Acute anxiolytic effects were not measured in this study, however general or pain-related anxiety relief may be an important mechanism behind both positive subjective responses to and behavioural demand for opioids. Future studies aiming to clinically attenuate or prevent risk of opioid use problems for people with childhood traumas could examine the importance of anxiety relief for driving drug use behaviour.

The drug effect questionnaire (DEQ) asked participants to rate how much they would like more of the drug they received *right now*, while the HPT asked them to report how many additional doses of this drug they would choose to *bring home*. This difference in immediate versus long-term need may have contributed to a disparity between the heightened self-reported wanting for high dose morphine, and the unaltered behavioural economic demand for this drug among the participants with childhood traumas. Research on both rodents and humans indicates that opioids are preferred in a safe and relaxing context [13]. There may be group differences in the extent to which participants’ home represents an opioid-compatible context, i.e., participants with childhood traumas may be less likely to consider their home a safe place to store or consume opioids, as they reported coming from a significantly less socially supportive environment compared to those without childhood trauma (Table 1). This context effect on opioid value could help explain why these participants were reluctant to bring high dose morphine home despite desiring more of this drug while situated in the research lab. It would also account for the reduced self-reported liking of opioids when administered in the stressful context of impending surgery [12]. Greater convergence with self-reported wanting in people with childhood traumas might have been observed if participants were asked to report their economic demand for a second dose in the same experimental context.

Moreover, the HPT was delivered while participants were still reporting feeling the effects of the high dose morphine (i.e., 31–51% of the median full effect; Fig. 1), and when negative drug effects such as nausea, dizziness and sedation were more likely [11]. Feeling psychoactive or negative drug effects may have dampened behavioural economic demand for this drug. Studies have shown that acutely feeling the effect (or feeling ‘satiated’) of a reinforcing commodity (e.g., sucrose [51] or alcohol [52]) will acutely reduce the desire to consume more. While feeling the effects were not particularly high when the HPT was delivered, and were less likely to be positive drug effects, it is nevertheless possible that demand for the high dose morphine would have more closely mirrored acute self-reported desires [11] and been different between participants with and without childhood traumas after the drug effects had fully dissipated. Further behavioural economic studies of opioid demand could explore differences in demand at peak positive drug effects, and address potential ‘satiety’ effects either observationally by measuring satiety, or experimentally by manipulating satiety to acute opioid effects.

The HPT used in this study assessed drug demand without imposing actual economic costs on the respondent or providing the respondents with the purchased drug. This may have led to high degree of ‘stockpiling’ at low prices, and likely limits the generalisability of point estimates of the consumption, expenditure, and demand indices reported here. Participants were also not made aware of the cost of a typical morphine dose during task instruction—a design choice that is not typical for HPT tasks, yet was selected for this study to assess the ‘personal value’ of the morphine dose in absence of a price estimate. This however likely led to greater variation in participant responses. To increase ecological validity, future studies using purchasing tasks to infer opioid misuse potential in people with childhood trauma could incorporate real costs and post-task drug administration (see e.g., Wilson et al. [53] and Amlung et al. [54]). This would help further bridge the gap between studies assessing risk of opioid misuse in people with childhood trauma (e.g., Carlyle et al. [11]) and observational studies estimating the prevalence of childhood trauma in people with opioid use disorder (see Santo et al. [7]).

A limitation of the current study is the absence of additional relevant social stress measures, including socioeconomic status (SES) or adult traumatic events, or additional resilience measures, such as prior psychosocial support or intervention. Considering the association between low SES and childhood trauma [55] and lower perceived social support in this study, future studies of opioid responses and behaviour in individuals with adverse childhood experiences should measure the impact of cumulative social stress factors that incorporate SES and broader living conditions, including education, employment, income, housing, social network, and social support. Furthermore, pairwise comparisons in the current analyses were not always qualified by significant interaction effects. This may have inflated the Type I error rate. However, the use of the conservative Bonferroni method for adjusting *p*-values in the pairwise comparisons, and the similar pattern observed across overall consumption and expenditure and specific demand indices, strengthen our confidence in the results. While this was a relatively small study in 47 individuals, the counterbalanced crossover design enabled each participant to serve as their own control. Nevertheless, more studies are needed to determine the robustness of our findings.

## Conclusion

Opioids are an important tool in pain management, yet are also highly addictive substances. Striking a balance between risk and relief is therefore vital to ensure patients’ long-term well-being. Screening tools have been developed to help clinicians identify patients who may require closer follow-up if treated with opioids for pain, including those with history of childhood trauma [56]. Unfortunately, the use of such tools has in some instances led to inadequate pain treatment, including the denial of opioid pain treatment to patients with adverse childhood experiences [57]. While self-reported liking of opioid drugs is considered one of *the* most important indicators of risk of misuse [17], and some prior evidence suggests that individuals with history of childhood trauma are likely to experience opioid drugs as rewarding [11], we find no conclusive evidence for these individuals being more inclined to purchase morphine than those without childhood traumas in the current study. Our findings suggest that under some conditions, desire to use opioids may not necessarily manifest in strong intentions to acquire these drugs. Identifying the conditions under which trauma-related differences in opioid value emerge may be key to developing preventions and ensuring good pain management in patients with childhood traumas, while minimising risk of prolonged opioid use and misuse.

## Supplementary Information

Supplementary Material 1.

## Data Availability

The study data is not publicly available due to restrictions in ethical approvals, due to protecting sensitive participant data. Data may be available on request by emailing the study authors and undergoing approvals.
