# Supplementary material for: Assessing behavioural economic demand for morphine after an acute dose in individuals with- and without childhood trauma: a double-blind, randomised, controlled trial
Source: Psychopharmacology (Berl). 2025 Dec 4;243(10):2277–90. doi: 10.1007/s00213-025-06977-7 (PMC12942358; doi:10.1007/s00213-025-06977-7)
Supplement: Supplementary file 1 [file 213_2025_6977_MOESM1_ESM.docx]

Supplementary materials

Assessing Behavioural Economic Demand for Morphine After an Acute Dose in Individuals With- and Without Childhood Trauma: A Double-Blind, Randomised, Controlled Trial

Martin Trøstheim, Benjamin L. Berey, Elizabeth R. Aston, Matthew J. Gullo, Lee Hogarth, Graham Simpson, Rupert Broomby, Celia J. Morgan, Molly Carlyle

**Contents**

[1	Hypothetical Purchase Task questionnaire	2](#_Toc214966722)

[2	Calculating demand indices from responses to the HPT	4](#_Toc214966723)

[3	Consumption, expenditure, and demand index distributions	4](#_Toc214966724)

[4	Implementation of non-parametric analyses in R	9](#_Toc214966725)

[4.1	Breakpoint imputation	9](#_Toc214966726)

[5	Correspondence between rank scores and original values	9](#_Toc214966727)

[6	Prior opioid experience	11](#_Toc214966728)

[7	Self-reported drug effects	12](#_Toc214966729)

[8	Hypothetical purchase task data quality	13](#_Toc214966730)

[9	Replication analysis: Self-reported drug wanting	14](#_Toc214966731)

[10	Effects of group and dose on self-reported drug wanting, consumption, expenditure, and demand indices	15](#_Toc214966732)

[11	Effects of dose order on self-reported drug wanting, consumption, expenditure, and demand indices	18](#_Toc214966733)

[12	Effects of prior opioid experience on self-reported drug wanting, consumption, expenditure, and demand indices	19](#_Toc214966734)

[13	Contributions of perceived social support, depression, anxiety, pain catastrophising and childhood trauma severity to demand	20](#_Toc214966735)

[References	22](#_Toc214966736)

# 1 Hypothetical Purchase Task questionnaire

The following questions ask how many doses of morphine you would choose to take home with you if they cost various amounts of money. The doses would be exactly the same as the one you received earlier. Assume that you have the same income/savings that you have now and NO ACCESS to any other opiate or painkiller products other than those offered at these prices. Note: please pay close attention to the prices and costs per dose for each item because they change by different amounts.

| 1. How many doses would you take home if they were | FREE? | _______ |
| --- | --- | --- |
| 2. How many doses would you take home if they were | 2p each? | _______ |
| 3. How many doses would you take home if they were | 4p each? | _______ |
| 4. How many doses would you take home if they were | 6p each? | _______ |
| 5. How many doses would you take home if they were | 8p each? | _______ |
| 6. How many doses would you take home if they were | 10p each? | _______ |
| 7. How many doses would you take home if they were | 12p each? | _______ |
| 8. How many doses would you take home if they were | 14p each? | _______ |
| 9. How many doses would you take home if they were | 16p each? | _______ |
| 10. How many doses would you take home if they were | 18p each? | _______ |
| 11. How many doses would you take home if they were | 20p each? | _______ |
| 12. How many doses would you take home if they were | 22p each? | _______ |
| 13. How many doses would you take home if they were | 24p each? | _______ |
| 14. How many doses would you take home if they were | 26p each? | _______ |
| 15. How many doses would you take home if they were | 28p each? | _______ |
| 16. How many doses would you take home if they were | 30p each? | _______ |
| 17. How many doses would you take home if they were | 32p each? | _______ |
| 18. How many doses would you take home if they were | 34p each? | _______ |
| 19. How many doses would you take home if they were | 36p each? | _______ |
| 20. How many doses would you take home if they were | 38p each? | _______ |
| 21. How many doses would you take home if they were | 40p each? | _______ |
| 22. How many doses would you take home if they were | 42p each? | _______ |
| 23. How many doses would you take home if they were | 44p each? | _______ |
| 24. How many doses would you take home if they were | 46p each? | _______ |
| 25. How many doses would you take home if they were | 48p each? | _______ |
| 26. How many doses would you take home if they were | 50p each? | _______ |
| 27. How many doses would you take home if they were | 52p each? | _______ |
| 28. How many doses would you take home if they were | 54p each? | _______ |
| 29. How many doses would you take home if they were | 56p each? | _______ |
| 30. How many doses would you take home if they were | 58p each? | _______ |
| 31. How many doses would you take home if they were | 60p each? | _______ |
| 32. How many doses would you take home if they were | 70p each? | _______ |
| 33. How many doses would you take home if they were | 80p each? | _______ |
| 34. How many doses would you take home if they were | 90p each? | _______ |
| 35. How many doses would you take home if they were | £1 each? | _______ |
| 36. How many doses would you take home if they were | £1.50 each? | _______ |
| 37. How many doses would you take home if they were | £2 each? | _______ |
| 38. How many doses would you take home if they were | £2.50 each? | _______ |
| 39. How many doses would you take home if they were | £3 each? | _______ |
| 40. How many doses would you take home if they were | £4 each? | _______ |
| 41. How many doses would you take home if they were | £5 each? | _______ |

# 2 Calculating demand indices from responses to the HPT

We derived the demand indices *Intensity*, *O_max_*, *P_max_*, *Breakpoint*, and *Elasticity* from the raw HPT data^[1]^. *Intensity* (i.e., the consumption when additional doses are free) was derived from the response to the first item on the HPT. By calculating the maximum product of the hypothetical price listed in the HPT and number of doses the participants indicated they would purchase at these prices, we obtained *O_max_* (i.e., the maximum expenditure on additional doses). *P_max_* (i.e., the price that maximises expenditure) was then operationalised as the maximum price listed in the HPT that resulted in *O_max_* when multiplied with the indicated number of doses. We obtained *Breakpoint* (i.e., the lowest price that suppresses consumption) by calculating the minimum price at which the participants reported they would purchase 0 additional doses^[1]^. Participants with no *Breakpoint* were assigned a value of £6 to be ranked highest in the analyses. Finally, we applied the midpoint method^[2]^ and computed the average *Elasticity* (i.e., the rate of change in consumption as price increases) along the observed consumption curve (**Equation 1**).

|  | **(1)** |
| --- | --- |

In **Equation 1**, *k* is the number of price points, *i* is the index of the presented price point and observed hypothetical consumption, and *Q_i_* and *Q_i_*_−1_ are the observed hypothetical consumption at presented price points *P_i_* and *P_i_*_−1_, respectively. Participants who reported no hypothetical consumption additional doses across all price paints were assigned an *Elasticity* value of 0.

# 3 Consumption, expenditure, and demand index distributions

Response distributions for individual HPT price points were notably skewed and contained several extreme outliers (**Table S1**). These properties carried over to the measures derived from the HPT, including *Consumption_AUC_* and *Expenditure_AUC_*, and the demand indices *Intensity*, *O_max_*, *P_max_*, *Breakpoint*, and *Elasticity* (**Table S2**). Due to the skewness and presence of outliers, the mean is less useful for describing the typical demand for the study drugs in the current sample (**Figure S1**). Median-based approaches such as non-parametric models are therefore better suited than mean-based parametric models for analysing the data.

**Table S1**

*Skewness and outliers in responses to each price point in the HPT.*

|  | **Consumption** | | **Expenditure** | |
| --- | --- | --- | --- | --- |
| **Price** | **Skewness** | **Outliers** | **Skewness** | **Outliers** |
| £0 | 7.59 | 9 (10%) | --- | 0 (0%) |
| £0.02 | 6.22 | 9 (10%) | 6.22 | 9 (10%) |
| £0.04 | 6.59 | 9 (10%) | 6.59 | 9 (10%) |
| £0.06 | 5.93 | 8 (9%) | 5.93 | 8 (9%) |
| £0.08 | 4.98 | 7 (7%) | 4.98 | 7 (7%) |
| £0.1 | 8.44 | 6 (6%) | 8.44 | 6 (6%) |
| £0.12 | 4.10 | 10 (11%) | 4.10 | 10 (11%) |
| £0.14 | 3.91 | 10 (11%) | 3.91 | 10 (11%) |
| £0.16 | 3.80 | 10 (11%) | 3.80 | 10 (11%) |
| £0.18 | 4.17 | 9 (10%) | 4.17 | 9 (10%) |
| £0.2 | 4.00 | 10 (11%) | 4.00 | 10 (11%) |
| £0.22 | 4.02 | 17 (18%) | 4.02 | 17 (18%) |
| £0.24 | 4.01 | 18 (19%) | 4.01 | 18 (19%) |
| £0.26 | 4.28 | 18 (19%) | 4.28 | 18 (19%) |
| £0.28 | 3.90 | 19 (20%) | 3.90 | 19 (20%) |
| £0.3 | 4.54 | 18 (19%) | 4.54 | 18 (19%) |
| £0.32 | 4.72 | 16 (17%) | 4.72 | 16 (17%) |
| £0.34 | 4.64 | 15 (16%) | 4.64 | 15 (16%) |
| £0.36 | 4.77 | 19 (20%) | 4.77 | 19 (20%) |
| £0.38 | 4.80 | 19 (20%) | 4.80 | 19 (20%) |
| £0.4 | 4.74 | 17 (18%) | 4.74 | 17 (18%) |
| £0.42 | 4.93 | 17 (18%) | 4.93 | 17 (18%) |
| £0.44 | 4.67 | 17 (18%) | 4.67 | 17 (18%) |
| £0.46 | 4.54 | 19 (20%) | 4.54 | 19 (20%) |
| £0.48 | 4.45 | 19 (20%) | 4.45 | 19 (20%) |
| £0.5 | 4.35 | 14 (15%) | 4.35 | 14 (15%) |
| £0.52 | 4.38 | 15 (16%) | 4.38 | 15 (16%) |
| £0.54 | 4.40 | 17 (18%) | 4.40 | 17 (18%) |
| £0.56 | 4.46 | 19 (20%) | 4.46 | 19 (20%) |
| £0.58 | 4.33 | 19 (20%) | 4.33 | 19 (20%) |
| £0.6 | 3.92 | 18 (19%) | 3.92 | 18 (19%) |
| £0.7 | 3.84 | 16 (17%) | 3.84 | 16 (17%) |
| £0.8 | 3.78 | 14 (15%) | 3.78 | 14 (15%) |
| £0.9 | 4.20 | 14 (15%) | 4.20 | 14 (15%) |
| £1 | 3.98 | 17 (18%) | 3.98 | 17 (18%) |
| £1.5 | 3.56 | 11 (12%) | 3.56 | 11 (12%) |
| £2 | 3.36 | 10 (11%) | 3.36 | 10 (11%) |
| £2.5 | 3.36 | 10 (11%) | 3.36 | 10 (11%) |
| £3 | 3.15 | 11 (12%) | 3.15 | 11 (12%) |
| £4 | 2.93 | 11 (12%) | 2.93 | 11 (12%) |
| £5 | 3.00 | 10 (11%) | 3.00 | 10 (11%) |

*Note*. N = 94 sessions. Outliers defined as > Q3+1.5×IQR or < Q1−1.5×IQR.

**Table S2**

*Properties of self-reported drug wanting, consumption, expenditure, and demand index distributions.*

|  | **Skewness** | **Outliers** | **No Intensity** | **No Pmax** | **No Breakpoint** | **Perfect inelasticity** |
| --- | --- | --- | --- | --- | --- | --- |
| **Want more** | 1.08 | 0 (0%) | --- | --- | --- | --- |
| **Consumption_AUC_** | 3.98 | 15 (16%) | --- | --- | --- | --- |
| **Expenditure_AUC_** | 3.09 | 11 (12%) | --- | --- | --- | --- |
| **Intensity** | 7.59 | 9 (10%) | 14 (15%) | --- | --- | --- |
| **O_max_** | 3.83 | 13 (14%) | --- | --- | --- | --- |
| **P_max_** | 0.46 | 0 (0%) | --- | 15 (16%) | --- | --- |
| **Breakpoint** | --- | --- | --- | --- | 53 (56%) | --- |
| **Elasticity** | 1.97 | 5 (5%) | --- | --- | --- | 20 (21%) |

*Note*. N = 94 sessions. Outliers defined as > Q3+1.5×IQR or < Q1−1.5×IQR. “No Intensity” indicates that participants did not want any free additional doses. “No Pmax” indicates that participants did not want to pay for any additional doses. “No breakpoint” indicates that participants were willing to pay more than the maximum price (£5) listed in the HPT per additional dose. “Perfect inelasticity” indicates that participants’ demand for additional doses did not change as a function of price. In 14 session (15%) there was no demand for the study drug.

**Figure S1.** Subjective drug wanting, consumption, expenditure, and demand index distributions presented as histograms (grey bars) and density curves (black curves). N indicates the number of sessions. Vertical lines indicate the mean (red) and the median (blue) of each distribution. *Consumption_AUC_*, *Expenditure_AUC_*, *Intensity* and *O_max_* are displayed on a cube root scale. Distributions, means and medians for *Breakpoint* are based on the original observed data (greydensity curve and solid vertical lines) in which a *Breakpoint* was reached in 41 sessions (44%), and on imputed data (yellow bar and dashed vertical lines) in which *Breakpoint* was set to 6 for the 53 (56%) sessions where participants did not reach a *Breakpoint*.

# 4 Implementation of non-parametric analyses in R

All non-parametric analyses were conducted in R^[3]^. We implemented non-parametric rank-based mixed models and tests of within-group changes in outcomes with the *f1.ld.f1* function from the *nparLD* package^[4]^. Fisher’s exact tests for between-group differences in sample characteristics were conducted with the function *fisher.exact* from the package *exact2x2*^[5–7]^. For Kruskal-Wallis *H*-tests of between-group differences in outcomes and sample characteristics, we used the *kruskal.test* function. The *p.adjust* function was used to apply the Bonferroni method for family-wise error rate (FWER) correction to p-values obtained omnibus tests of multiple outcomes and post-hoc pairwise comparisons. We computed Stuart-Kendall Tau-c rank-based correlations between outcomes with the *StuartTauC* function from the package *DescTools*^[8]^.

## 4.1 Breakpoint imputation

Participants did not reach a *Breakpoint* in 53 (56%) sessions, indicating that their *Breakpoints* for the study drugs are greater than £5. To ensure that these participants were ranked the highest in the non-parametric analyses, we assigned them a *Breakpoint* value of 6. Descriptive statistics for *Breakpoint* after this imputation are available in **Table S3**.

**Table S3**

*Descriptive statistics for Breakpoint after imputation.*

|  | **Control (n = 21)** | | **Childhood trauma (n = 26)** | |
| --- | --- | --- | --- | --- |
|  | **High dose** | **Low dose** | **High dose** | **Low dose** |
| **Breakpoint (£)** |  |  |  |  |
| Mean (SD) | 4.48 (2.50) | 4.45 (2.41) | 4.20 (2.60) | 2.36 (2.53) |
| Median (IQR) | 6.00 (2.50-6.00) | 6.00 (3.00-6.00) | 6.00 (1.62-6.00) | 1.50 (0.00-5.50) |
| Range | 0.00-6.00 | 0.00-6.00 | 0.00-6.00 | 0.00-6.00 |

*Note. Breakpoint* = 6 was assigned to all participants who did not reach a *Breakpoint* to ensure that they received the highest rank in the non-parametric, rank-based analyses.

# 5 Correspondence between rank scores and original values

The primary analyses were all non-parametric and rank-based. **Figure S2** illustrates how *Want more*, *Consumption_AUC_* and *Expenditure_AUC_,* and the demand indices *Intensity*, *O_max_*, *P_max_*, *Breakpoint* and *Elasticity* were converted to rank scores within these analyses.

**Figure S2**. Correspondence between rank scores and original values. Ties were accounted for by averaging ranks. *Consumption_AUC_*, *Expenditure_AUC_*, *Intensity* and *O_max_* are displayed on a cube root scale.

# 6 Prior opioid experience

All participants were asked whether they had ever been medically administered morphine or other opioids in the past, and whether they had taken non-medical opioids recreationally in the past (all required a yes/no response; **Table S4**).

**Table S4**

*Descriptive statistics for prior opioid experience.*

|  | **Control (n = 21)** | **Childhood trauma (n = 26)** |  |
| --- | --- | --- | --- |
| **Prior opioid experience** |  |  | *p* = 0.24 |
| Yes | 7 (33%) | 14 (54%) |  |
| No | 14 (67%) | 12 (46%) |  |
| **Prior licit opioid experience** |  |  | *p* = 0.37 |
| Yes | 7 (67%) | 13 (50%) |  |
| No | 14 (67%) | 13 (50%) |  |
| **Prior morphine experience** |  |  | *p* = 0.76 |
| Yes | 6 (29%) | 9 (35%) |  |
| No | 15 (71%) | 17 (65%) |  |
| **Prior illicit opioid experience** |  |  | *p* = 1 |
| Yes | 1 (5%) | 2 (8%) |  |
| No | 20 (95%) | 24 (92%) |  |

*Note*. Prior opioid experience was defined as having used morphine, other medical opioids, or non-medical opioids in the past. Prior licit opioid experience was defined as past medical use of morphine or other opioids. Prior morphine experience was defined as past medical use of morphine. Finally, prior illicit opioid experience was defined as past recreational use of non-medical opioids. Differences between groups were assessed with Fisher’s exact tests.

# 7 Self-reported drug effects

In addition to reporting their wanting for the study drug (*Want more*; “Would you like MORE of the drug you took, right now?”), participants also rated how much they felt the drug effect (*Feel effect*; “Do you FEEL a drug effect right now?”), their experience of drug high (*High*; “Are you HIGH right now?”), and their liking (*Like*; “Do you LIKE any of the effects you are feeling right now?”) and disliking (*Dislike*; “Do you DISLIKE any of the effects you are feeling right now?”), of the drug effects they were experiencing. All items were from the Drug Effects Questionnaire (DEQ)^[9]^ and were rated on a 0-100 visual analogue scale (VAS; 0 = “not at all”, 100 = “extremely”) 15, 30, 45, 60, 90, 120 and 150 minutes after drug administration. Descriptive statistics for these self-reported drug effects leading up to the hypothetical purchase task (i.e., 15-120 minutes after drug administration) are available in **Table S5**.

**Table S5**

*Descriptive statistics for self-reported drug effects leading up to the HPT.*

|  | **Control (n = 21)** | | **Childhood trauma (n = 26)** | |
| --- | --- | --- | --- | --- |
|  | **High dose** | **Low dose** | **High dose** | **Low dose** |
| **Feel effect (0-100)** |  |  |  |  |
| Mean (SD) | 36 (25) | 10 (10) | 35 (27) | 8 (13) |
| Median (IQR) | 33 (13-60) | 11 (0-16) | 30 (13-52) | 1 (0-11) |
| Range | 4-77 | 0-30 | 0-92 | 0-54 |
| **High (0-100)** |  |  |  |  |
| Mean (SD) | 19 (22) | 6 (11) | 23 (21) | 6 (10) |
| Median (IQR) | 13 (3-25) | 0 (0-10) | 16 (6-33) | 1 (0-8) |
| Range | 0-76 | 0-47 | 0-76 | 0-35 |
| **Like (0-100)** |  |  |  |  |
| Mean (SD) | 23 (18) | 20 (24) | 39 (25) | 24 (22) |
| Median (IQR) | 19 (12-36) | 6 (0-47) | 42 (20-52) | 18 (2-50) |
| Range | 0-55 | 0-72 | 2-98 | 0-64 |
| **Dislike (0-100)** |  |  |  |  |
| Mean (SD) | 22 (22) | 9 (15) | 14 (22) | 7 (14) |
| Median (IQR) | 15 (3-43) | 1 (0-9) | 4 (0-17) | 0 (0-6) |
| Range | 0-78 | 0-50 | 0-90 | 0-50 |
| **Want more (0-100)** |  |  |  |  |
| Mean (SD) | 7 (12) | 7 (13) | 40 (31) | 19 (22) |
| Median (IQR) | 0 (0-7) | 0 (0-7) | 48 (4-62) | 9 (0-38) |
| Range | 0-44 | 0-50 | 0-91 | 0-66 |

*Note.* SD = standard deviation. IQR = interquartile range. Self-reported drug effects (*Feel effect*, *High*, *Like*, *Dislike*, and *Want more*) were rated on a 0-100 VAS at 15, 30, 45, 60, 90, and 120 minutes after drug administration and are expressed as the percentage of the total possible AUC (10500).

# 8 Hypothetical purchase task data quality

The quality of the HPT data was determined by the absence of increasing demand with increasing price and is summarised in **Table S6**.

**Table S6**

*Hypothetical purchase task data quality.*

|  | **Control (n = 21)** | | **Childhood trauma (n = 26)** | |
| --- | --- | --- | --- | --- |
|  | **High dose** | **Low dose** | **High dose** | **Low dose** |
| **Bounces** |  |  |  |  |
| Mean (SD) | 0.10 (0.30) | 0.24 (0.54) | 0.12 (0.33) | 0.23 (0.59) |
| Median (IQR) | 0 (0-0) | 0 (0-0) | 0 (0-0) | 0 (0-0) |
| Range | 0-1 | 0-2 | 0-1 | 0-2 |
| **Reversals from zero** |  |  |  |  |
| Mean (SD) | 0 (0) | 0 (0) | 0 (0) | 0 (0) |
| Median (IQR) | 0 (0-0) | 0 (0-0) | 0 (0-0) | 0 (0-0) |
| Range | 0-0 | 0-0 | 0-0 | 0-0 |
| **Trends** |  |  |  |  |
| Mean (SD) | 504 (2177) | 1025 (4351) | 15 (40) | 8 (21) |
| Median (IQR) | 4 (1-19) | 10 (1-20) | 2 (0-15) | 1 (0-6) |
| Range | 0-9996 | 0-19996 | 0-198 | 0-100 |

*Note*. Bounces indicate the number of times non-zero consumption increased with increasing price. Reversals from zero indicate the number of times consumption increased from zero to non-zero with increasing price. Trends indicate the decrease in consumption per £1 increase in price and was calculated as (Consumption_Lowest price_ − Consumption_Highest price_) ∕ (Highest price − Lowest price).

# 9 Replication analysis: Self-reported drug wanting

The pattern of self-reported drug wanting (*Want more*) in the analytic sample (**Table 2**) mirrored that in the complete sample^[10]^. There was a significant main effect of *Group* (*ATS*_1_ = 14.79, *p* < 0.001) and *Dose* (*ATS*_1_ = 7.18, *p* < 0.01), and a significant *Group*×*Dose* interaction effect (*ATS*_1_ = 6.30, *p* = 0.01) on *Want more*. Post-hoc pairwise comparisons indicated greater *Want more* for high dose morphine (Kruskal-Wallis *H*_1_ = 14.08, *p*_adjusted_ < 0.001), but not low dose morphine (Kruskal-Wallis *H*_1_ = 3.50, *p*_adjusted_ = 0.25) in the childhood trauma group compared than in the control group. Moreover, *Want more* was greater for high dose morphine than for low dose morphine in the childhood trauma group (*ATS*_1_ = 11.96, *p*_adjusted_ < 0.01), but not in the control group (*ATS*_1_ = 0.02, *p*_adjusted_ = 1).

# 10 Effects of group and dose on self-reported drug wanting, consumption, expenditure, and demand indices

An overview of results from rank-based non-parametric analyses assessing the effects of *Group* and *Dose* on self-reported drug wanting, consumption and expenditure, and demand indices is presented in **Table S7**. Based on these models, we calculated the average rank and relative treatment effect (RTE) for each *Group*×*Dose* combination (**Table S8**). **Table S9** details the results from follow-up post-hoc pairwise comparisons.

**Table S7**

*Effects of Group (childhood trauma vs. control) and Dose (high dose vs. low dose) on self-reported drug wanting, consumption, expenditure, and demand indices.*

|  | **ATS (df)** | **p** | **p_adjusted_** |
| --- | --- | --- | --- |
| **Want more** |  |  |  |
| Group | 14.79 (1) | < 0.001 | < 0.001 |
| Dose | 7.18 (1) | < 0.01 | < 0.01 |
| Group×Dose | 6.30 (1) | 0.01 | 0.01 |
| **Consumption_AUC_** |  |  |  |
| Group | 4.69 (1) | 0.03 | 0.06 |
| Dose | 7.12 (1) | < 0.01 | 0.02 |
| Group×Dose | 4.23 (1) | 0.04 | 0.08 |
| **Expenditure_AUC_** |  |  |  |
| Group | 5.33 (1) | 0.02 | 0.04 |
| Dose | 10.50 | < 0.01 | < 0.01 |
| Group×Dose | 5.09 | 0.02 | < 0.05 |
| **Intensity** |  |  |  |
| Group | 3.94 (1) | < 0.05 | 0.24 |
| Dose | 0.79 (1) | 0.37 | 1 |
| Group×Dose | 3.48 (1) | 0.06 | 0.31 |
| **O_max_** |  |  |  |
| Group | 4.55 (1) | 0.03 | 0.16 |
| Dose | 7.65 (1) | < 0.01 | 0.03 |
| Group×Dose | 5.02 (1) | 0.03 | 0.13 |
| **P_max_** |  |  |  |
| Group | 2.35 (1) | 0.13 | 0.63 |
| Dose | 8.43 (1) | < 0.01 | 0.02 |
| Group×Dose | 1.29 (1) | 0.26 | 1 |
| **Breakpoint** |  |  |  |
| Group | 3.67 (1) | 0.06 | 0.28 |
| Dose | 11.63 (1) | < 0.001 | < 0.01 |
| Group×Dose | 9.56 (1) | < 0.01 | < 0.01 |
| **Elasticity** |  |  |  |
| Group | 0.23 (1) | 0.63 | 1 |
| Dose | 1.10 (1) | 0.29 | 1 |
| Group×Dose | 3.33 (1) | 0.07 | 0.34 |

*Note.* *p*-values were adjusted for multiple tests with the Bonferroni method. *Want more*: n = 1 test. *Consumption_AUC_* and *Expenditure_AUC_*: n = 2 tests. *Intensity*, *O_max_*, *P_max_*, *Breakpoint* and *Elasticity*: n = 5 tests.

**Table S8**

*Average rank and relative treatment effect of each Group×Dose combination.*

|  | **Control (n = 21)** | | **Childhood trauma (n = 26)** | |
| --- | --- | --- | --- | --- |
|  | **High dose** | **Low dose** | **High dose** | **Low dose** |
| **Want more** |  |  |  |  |
| Average rank | 35.48 | 34.88 | 66.54 | 48.37 |
| RTE [95% CI] | 0.37 [0.30-0.46] | 0.37 [0.29-0.45] | 0.70 [0.62-0.76] | 0.51 [0.43-0.58] |
| **Consumption_AUC_** |  |  |  |  |
| Average rank | 56.98 | 55.21 | 47.35 | 33.77 |
| RTE [95% CI] | 0.60 [0.51-0.68] | 0.58 [0.49-0.66] | 0.50 [0.41-0.59] | 0.35 [0.28-0.44] |
| **Expenditure_AUC_** |  |  |  |  |
| Average rank | 57.98 | 55.19 | 47.94 | 32.38 |
| RTE [95% CI] | 0.61 [0.52-0.69] | 0.58 [0.49-0.66] | 0.50 [0.42-0.59] | 0.34 [0.27-0.42] |
| **Intensity** |  |  |  |  |
| Average rank | 54.17 | 56.79 | 44.75 | 37.37 |
| RTE [95% CI] | 0.57 [0.48-0.65] | 0.60 [0.51-0.68] | 0.47 [0.39-0.56] | 0.39 [0.32-0.48] |
| **O_max_** |  |  |  |  |
| Average rank | 56.60 | 54.93 | 48.77 | 32.88 |
| RTE [95% CI] | 0.60 [0.51-0.67] | 0.58 [0.49-0.66] | 0.51 [0.42-0.60] | 0.34 [0.28-0.43] |
| **P_max_** |  |  |  |  |
| Average rank | 56.60 | 49.19 | 51.60 | 34.69 |
| RTE [95% CI] | 0.60 [0.49-0.69] | 0.52 [0.42-0.62] | 0.54 [0.46-0.62] | 0.36 [0.30-0.45] |
| **Breakpoint** |  |  |  |  |
| Average rank | 54.48 | 53.57 | 51.46 | 33.00 |
| RTE [95% CI] | 0.57 [0.49-0.65] | 0.56 [0.48-0.64] | 0.54 [0.47-0.61] | 0.35 [0.28-0.42] |
| **Elasticity** |  |  |  |  |
| Average rank | 44.38 | 54.38 | 47.33 | 44.63 |
| RTE [95% CI] | 0.47 [0.38-0.56] | 0.57 [0.48-0.65] | 0.50 [0.41-0.58] | 0.47 [0.37-0.57] |

*Note*. Relative treatment effect (RTE) indicates the probability that a randomly drawn observation from the entire sample has lower *Want more*, *Consumption_AUC_*, *Expenditure_AUC_*, *Intensity*, *O_max_*, *P_max_*, *Breakpoint* or *Elasticity* than a randomly drawn observation from the particular *Group*×*Dose* combination for which the RTE was estimated.

**Table S9**

*Parametric post-hoc comparisons of self-reported drug wanting, consumption, expenditure, and demand indices between doses within groups, and between groups within doses.*

|  | **H (df) or ATS (df)** | **p** | **p_adjusted_** |
| --- | --- | --- | --- |
| **Want more** |  |  |  |
| Trauma (low dose) vs. Control (low dose) | 3.50 (1) | 0.06 | 0.25 |
| Trauma (high dose) vs. Control (high dose) | 14.08 (1) | < 0.001 | < 0.001 |
| Trauma (low dose) vs. Trauma (high dose) | 11.96 (1) | < 0.001 | < 0.01 |
| Control (low dose) vs. Control (high dose) | 0.02 (1) | 0.90 | 1 |
| **Consumption_AUC_** |  |  |  |
| Trauma (low dose) vs. Control (low dose) | 6.98 (1) | < 0.01 | 0.03 |
| Trauma (high dose) vs. Control (high dose) | 1.91 (1) | 0.17 | 0.67 |
| Trauma (low dose) vs. Trauma (high dose) | 7.00 (1) | < 0.01 | 0.03 |
| Control (low dose) vs. Control (high dose) | 0.46 (1) | 0.50 | 1 |
| **Expenditure_AUC_** |  |  |  |
| Trauma (low dose) vs. Control (low dose) | 8.12 (1) | < 0.01 | 0.02 |
| Trauma (high dose) vs. Control (high dose) | 2.03 (1) | 0.15 | 0.62 |
| Trauma (low dose) vs. Trauma (high dose) | 10.48 (1) | < 0.01 | < 0.01 |
| Control (low dose) vs. Control (high dose) | 0.87 (1) | 0.35 | 1 |
| **Intensity** |  |  |  |
| Trauma (low dose) vs. Control (low dose) | 5.75 (1) | 0.02 | 0.07 |
| Trauma (high dose) vs. Control (high dose) | 1.47 (1) | 0.23 | 0.90 |
| Trauma (low dose) vs. Trauma (high dose) | 2.11 (1) | 0.15 | 0.58 |
| Control (low dose) vs. Control (high dose) | 2.38 (1) | 0.12 | 0.49 |
| **O_max_** |  |  |  |
| Trauma (low dose) vs. Control (low dose) | 6.83 (1) | < 0.01 | 0.04 |
| Trauma (high dose) vs. Control (high dose) | 1.29 (1) | 0.26 | 1 |
| Trauma (low dose) vs. Trauma (high dose) | 8.56 (1) | < 0.01 | 0.01 |
| Control (low dose) vs. Control (high dose) | 0.26 (1) | 0.61 | 1 |
| **P_max_** |  |  |  |
| Trauma (low dose) vs. Control (low dose) | 3.56 (1) | 0.06 | 0.24 |
| Trauma (high dose) vs. Control (high dose) | 0.40 (1) | 0.53 | 1 |
| Trauma (low dose) vs. Trauma (high dose) | 11.60 (1) | < 0.001 | < 0.01 |
| Control (low dose) vs. Control (high dose) | 1.21 (1) | 0.27 | 1 |
| **Breakpoint** |  |  |  |
| Trauma (low dose) vs. Control (low dose) | 7.86 (1) | < 0.01 | 0.02 |
| Trauma (high dose) vs. Control (high dose) | 0.22 (1) | 0.64 | 1 |
| Trauma (low dose) vs. Trauma (high dose) | 18.60 (1) | < 0.0001 | < 0.0001 |
| Control (low dose) vs. Control (high dose) | 0.06 (1) | 0.81 | 1 |
| **Elasticity** |  |  |  |
| Trauma (low dose) vs. Control (low dose) | 1.21 (1) | 0.27 | 1 |
| Trauma (high dose) vs. Control (high dose) | 0.17 (1) | 0.68 | 1 |
| Trauma (low dose) vs. Trauma (high dose) | 0.18 (1) | 0.67 | 1 |
| Control (low dose) vs. Control (high dose) | 12.59 (1) | < 0.001 | < 0.01 |

*Note*. Rank-based comparisons between groups within doses were assessed with ANOVA-type statistics (*ATS*) tests. Rank-based comparisons between doses within groups were assessed with Kruskal-Wallis *H*-tests. *p*-values were adjusted for multiple pairwise comparisons with the Bonferroni method (n = 4 comparisons).

# 11 Effects of dose order on self-reported drug wanting, consumption, expenditure, and demand indices

Due to the randomised crossover design of this study, 26 (55%) of the participants in the analytic sample received low dose morphine before high dose morphine, while 21 (45%) received high dose morphine before low dose morphine. We conducted rank-based non-parametric analyses to assess whether *Dose order* affected participants’ self-reported drug wanting, consumption and expenditure, and demand indices. Results from these analyses are presented in **Table S10**.

**Table S10**

*Effects of Dose order (low dose-high dose vs. high dose-low dose) and Dose (high dose vs. low dose) on consumption, expenditure, and demand indices.*

|  | **ATS (df)** | **p** |
| --- | --- | --- |
| **Want more** |  |  |
| Dose order | 1.22 (1) | 0.27 |
| Dose | 7.97 (1) | < 0.01 |
| Dose order×Dose | 2.15 (1) | 0.14 |
| **Consumption_AUC_** |  |  |
| Dose order | 0.53 (1) | 0.47 |
| Dose | 6.32 (1) | 0.01 |
| Dose order×Dose | 0.01 (1) | 0.93 |
| **Expenditure_AUC_** |  |  |
| Dose order | 0.34 (1) | 0.56 |
| Dose | 9.66 (1) | < 0.01 |
| Dose order×Dose | 0.52 (1) | 0.47 |
| **Intensity** |  |  |
| Dose order | 0.74 (1) | 0.39 |
| Dose | 0.79 (1) | 0.37 |
| Dose order×Dose | 0.51 (1) | 0.47 |
| **O_max_** |  |  |
| Dose order | 0.28 (1) | 0.60 |
| Dose | 6.93 (1) | < 0.01 |
| Dose order×Dose | 0.08 (1) | 0.77 |
| **P_max_** |  |  |
| Dose order | 0.40 (1) | 0.53 |
| Dose | 10.78 (1) | < 0.01 |
| Dose order×Dose | 1.97 (1) | 0.16 |
| **Breakpoint** |  |  |
| Dose order | 0.40 (1) | 0.53 |
| Dose | 12.21 (1) | < 0.001 |
| Dose order×Dose | 1.32 (1) | 0.25 |
| **Elasticity** |  |  |
| Dose order | 0.60 (1) | 0.44 |
| Dose | 0.77 (1) | 0.38 |
| Dose order×Dose | 0.51 (1) | 0.48 |

*Note.* All *p*-values are unadjusted.

# 12 Effects of prior opioid experience on self-reported drug wanting, consumption, expenditure, and demand indices

Because 21 (45%) of the participants in the analytic had prior experience with using opioids, we conducted rank-based non-parametric analyses to determine whether *Prior opioid experience* influenced participants’ self-reported drug wanting, consumption and expenditure, and demand indices. Results from these analyses are presented in **Table S11**.

**Table S11**

*Effects of Prior opioid experience (yes vs. no) and Dose (high dose vs. low dose) on consumption, expenditure, and demand indices.*

|  | **ATS (df)** | **p** |
| --- | --- | --- |
| **Want more** |  |  |
| Prior opioid experience | < 0.01 (1) | 0.96 |
| Dose | 7.42 (1) | < 0.01 |
| Prior opioid experience×Dose | 0.08 (1) | 0.78 |
| **Consumption_AUC_** |  |  |
| Prior opioid experience | 0.07 (1) | 0.79 |
| Dose | 6.98 (1) | < 0.01 |
| Prior opioid experience×Dose | 2.13 (1) | 0.14 |
| **Expenditure_AUC_** |  |  |
| Prior opioid experience | 0.07 (1) | 0.79 |
| Dose | 10.44 (1) | < 0.01 |
| Prior opioid experience×Dose | 2.04 (1) | 0.15 |
| **Intensity** |  |  |
| Prior opioid experience | 0.95 (1) | 0.33 |
| Dose | 1.09 (1) | 0.30 |
| Prior opioid experience×Dose | 1.53 (1) | 0.22 |
| **O_max_** |  |  |
| Prior opioid experience | < 0.01 (1) | 0.97 |
| Dose | 7.59 (1) | < 0.01 |
| Prior opioid experience×Dose | 1.63 (1) | 0.20 |
| **P_max_** |  |  |
| Prior opioid experience | 2.07 (1) | 0.15 |
| Dose | 10.96 (1) | < 0.001 |
| Prior opioid experience×Dose | 2.67 (1) | 0.10 |
| **Breakpoint** |  |  |
| Prior opioid experience | 0.38 (1) | 0.54 |
| Dose | 12.96 (1) | < 0.001 |
| Prior opioid experience×Dose | 3.82 (1) | 0.05 |
| **Elasticity** |  |  |
| Prior opioid experience | 2.02 (1) | 0.16 |
| Dose | 0.38 (1) | 0.54 |
| Prior opioid experience×Dose | 1.08 (1) | 0.30 |

*Note.* All *p*-values are unadjusted.

# 13 Contributions of perceived social support, depression, anxiety, pain catastrophising and childhood trauma severity to demand

In addition to greater childhood trauma severity, the childhood trauma group reported significantly lower perceived social support and higher depression, anxiety and pain catastrophising than controls (**Table 1**). We therefore calculated rank-based correlation coefficients (Stuart-Kendall *Tau-c*) for the relationship between these characteristics and each demand measure across the two groups to explore whether the observed group differences in demand may be driven by differences in these characteristics rather than history of childhood trauma. The correlations were generally low (*Tau-c* = −0.09 to 0.22) and non-significant (**Table S13**). The only exception to this was that breakpoint for the high dose of morphine was significantly but weakly positively correlated with both anxiety (*Tau-c* = 0.22, *p*_unadjusted_ < 0.01) and pain catastrophising (*Tau-c* = 0.16, *p*_unadjusted_ < 0.05; **Table S13**). Importantly (and consistent with our main findings), childhood trauma severity was significantly and negatively correlated with several measures of demand for the low dose of morphine across groups, including overall consumption (*Tau-c* = −0.26, *p* = 0.01) and expenditure (−0.27, *p* < 0.01), intensity (−0.22, *p* = 0.02), O_max_ (*Tau-c* = −0.23, *p* = 0.01), P_max_ (*Tau-c* = −0.17, *p* = 0.03) and breakpoint (*Tau-c* = −0.24, *p* = 0.01).

**Table S12**

*Rank-based correlations (Stuart-Kendall Tau-c) between perceived social support, depression, anxiety, pain catastrophising and childhood trauma severity, and drug consumption, expenditure and demand indices.*

|  | **High dose** | **Low dose** |
| --- | --- | --- |
| **MSPSS** |  |  |
| Consumption_AUC_ | 0.00 (*p* = 0.97) | 0.08 (*p* = 0.47) |
| Expenditure_AUC_ | 0.00 (*p* = 0.96) | 0.11 (*p* = 0.27) |
| Intensity | −0.06 (*p* = 0.65) | 0.01 (*p* = 0.92) |
| O_max_ | 0.00 (*p* = 0.97) | 0.09 (*p* = 0.36) |
| P_max_ | 0.10 (*p* = 0.33) | 0.17 (*p* = 0.07) |
| Breakpoint | 0.01 (*p* = 0.87) | 0.14 (*p* = 0.17) |
| Elasticity | −0.09 (*p* = 0.37) | −0.05 (*p* = 0.68) |
| **DASS Anxiety** |  |  |
| Consumption_AUC_ | 0.08 (*p* = 0.39) | 0.04 (*p* = 0.72) |
| Expenditure_AUC_ | 0.11 (*p* = 0.25) | 0.03 (*p* = 0.72) |
| Intensity | 0.05 (*p* = 0.59) | −0.00 (*p* = 0.99) |
| O_max_ | 0.06 (*p* = 0.56) | 0.04 (*p* = 0.68) |
| P_max_ | 0.14 (*p* = 0.18) | 0.06 (*p* = 0.60) |
| Breakpoint | 0.22 (*p* = 0.01) | 0.07 (*p* = 0.48) |
| Elasticity | 0.09 (*p* = 0.36) | −0.02 (*p* = 0.83) |
| **DASS Depression** |  |  |
| Consumption_AUC_ | 0.11 (*p* = 0.25) | −0.01 (*p* = 0.89) |
| Expenditure_AUC_ | 0.09 (*p* = 0.82) | −0.02 (*p* = 0.85) |
| Intensity | 0.11 (*p* = 0.24) | −0.04 (*p* = 0.64) |
| O_max_ | 0.12 (*p* = 0.21) | −0.00 (*p* = 0.97) |
| P_max_ | −0.01 (*p* = 0.93 | −0.06 (*p* = 0.54 |
| Breakpoint | 0.06 (*p* = 0.47) | −0.03 (*p* = 0.77) |
| Elasticity | 0.09 (*p* = 0.41) | −0.09 (*p* = 0.44) |
| **PCS** |  |  |
| Consumption_AUC_ | 0.11 (*p* = 0.27) | −0.01 (*p* = 0.95) |
| Expenditure_AUC_ | 0.14 (*p* = 0.16) | −0.04 (*p* = 0.68) |
| Intensity | 0.15 (*p* = 0.14) | 0.01 (*p* = 0.54) |
| O_max_ | 0.11 (*p* = 0.26) | −0.03 (*p* = 0.74) |
| P_max_ | 0.16 (*p* = 0.12) | −0.04 (*p* = 0.68) |
| Breakpoint | 0.16 (*p* < 0.05) | −0.04 (*p* = 0.66) |
| Elasticity | 0.16 (*p* = 0.09) | 0.16 (*p* = 0.08) |
| **CTQ** |  |  |
| Consumption_AUC_ | −0.11 (p = 0.28) | −0.26 (p = 0.01) |
| Expenditure_AUC_ | −0.11 (p = 0.22) | −0.27 (p < 0.01) |
| Intensity | −0.12 (p = 0.23) | −0.22 (p = 0.02) |
| O_max_ | −0.09 (p = 0.37) | −0.23 (p = 0.01) |
| P_max_ | 0.01 (p = 0.89) | −0.17 (p = 0.03) |
| Breakpoint | 0.02 (p = 0.77) | −0.24 (p = 0.01) |
| Elasticity | 0.01 (p = 0.92) | −0.08 (p = 0.46) |

*Note.* MSPSS = Multidimensional Scale of Perceived Social Support. DASS = Depression Anxiety Stress Scales. PCS = Pain Catastrophizing Scale. CTQ = Childhood Trauma Questionnaire. All *p*-values are unadjusted.

# References

1.	Strickland JC, Lacy RT. Behavioral economic demand as a unifying language for addiction science: Promoting collaboration and integration of animal and human models. Experimental and Clinical Psychopharmacology 2020;28(4):404–16.

2.	Allen RGD. The Concept of Arc Elasticity of Demand: I. Rev Econ Stud 1934;1(3):226–9.

3.	R Core Team. R: A Language and Environment for Statistical Computing [Internet]. 2023;Available from: https://www.R-project.org/

4.	Noguchi K, Gel YR, Brunner E, Konietschke F. nparLD: An R Software Package for the Nonparametric Analysis of Longitudinal Data in Factorial Experiments. J Stat Softw 2012;50(12):1–23.

5.	Fay MP. Two-sided Exact Tests and Matching Confidence Intervals for Discrete Data. R J 2010;2(1):53–8.

6.	Fay MP. Confidence intervals that match Fisher’s exact or Blaker’s exact tests. Biostatistics 2010;11(2):373–4.

7.	Fay MP, Hunsberger SA, Nason M, Gabriel E, Lumbard K. exact2x2: Exact Tests and Confidence Intervals for 2x2 Tables [Internet]. 2023;Available from: https://cran.r-project.org/package=exact2x2

8.	Signorell A, Aho K, Alfons A, Anderegg N, Aragon T, Arachchige C, et al. DescTools: Tools for Descriptive Statistics [Internet]. 2023;Available from: https://cran.r-project.org/package=DescTools

9.	Morean ME, de Wit H, King AC, Sofuoglu M, Rueger SY, O’Malley SS. The drug effects questionnaire: psychometric support across three drug types. Psychopharmacology 2013;227(1):177–92.

10.	Carlyle M, Broomby R, Simpson G, Hannon R, Fawaz L, Mollaahmetoglu OM, et al. A randomised, double-blind study investigating the relationship between early childhood trauma and the rewarding effects of morphine. Addict Biol 2021;26(6):e13047.
